# Comparison of Allogeneic Stem Cell Transplantation and Non-Transplant Approaches in Elderly Patients with Advanced Myelodysplastic Syndrome: Optimal Statistical Approaches and a Critical Appraisal of Clinical Results Using Non-Randomized Data

**DOI:** 10.1371/journal.pone.0074368

**Published:** 2013-10-07

**Authors:** Ronald Brand, Hein Putter, Anja van Biezen, Dietger Niederwieser, Rodrigo Martino, Ghulam Mufti, Francesco Onida, Argiris Symeonidis, Christoph Schmid, Laurent Garderet, Marie Robin, Michel van Gelder, Jürgen Finke, Martin Bornhäuser, Guido Kobbe, Ulrich Germing, Theo de Witte, Nicolaus Kröger

**Affiliations:** 1 Department of Statistics, University Medical Center, Leiden, The Netherlands; 2 Department of Hematology/Oncology, University Medical Center, Leipzig, Germany; 3 Hospital Santa Creu I Sant Pau, Barcelona, Spain; 4 GKT School of Medicine, London, United Kingdom; 5 Fondazione IRCCS Ospedale Maggiore, University Medical Center, Milan, Italy; 6 Department of Hematology, Universitiy Medical Center, Patras, Greece; 7 Department of Hematology/Oncology, Augsburg, Germany; 8 Department of Hematology, Hopital St Antoine, Paris, France; 9 Department of BMT, Hopital St Louis, Paris, France; 10 Department of Hematology, University Maastricht, Maastricht, The Netherlands; 11 Department of Hematology/Oncology, University Medical Center, Freiburg, Germany; 12 Department of Hematology/Oncology, University Mediacal Center, Dresden, Germany; 13 Department of Hematology and Oncology, University Hospital, Düsseldorf, Germany; 14 Nijmegen Center for Life Sciences, Radboud University Medical Center, Nijmegen, The Netherlands; 15 Department for Stem Cell Transplantation, University Medical Center, Hamburg, Germany; Cardiff University, United Kingdom

## Abstract

Allogeneic stem cell transplantation (ASCT) from related or unrelated donors may cure patients with myelodysplastic syndromes (MDS), a heterogeneous group of clonal stem cell disorders. We analysed 384 elderly patients (55-69 years) with advanced MDS who received either ASCT (n=247) and were reported to The European Group for Blood and Marrow Transplantation (EBMT) or a non –transplant approach (n=137) reported to the Düsseldorf registry. Besides an attempt to answer the question of „comparison“, the purpose of this work is to explain the difficulties in comparing a non-transplant with a transplant cohort, when death before transplant is likely but unknown and the selection of patients for transplant is based on assumptions. It shows which methods are almost always biased and that even the most sophisticated approaches crucially rely on clinical assumptions. Using the most appropriate model for our data, we derive an overall univariate non-significant survival disadvantage for the transplant cohort (HR: 1.29, p = 0.11). We show that such an “average” hazard ratio is however misleading due to non-proportionality of the hazards reflecting early treatment related mortality, the occurring of which is logically correlated with the interval between diagnosis and transplant creating a disproportional drop in the (reconstructed) survival curve of the transplanted patients. Also in multivariate analysis (correcting for age > 60 (HR: 1.4, p = 0.02) and abnormal cytogenetics (HR: 1.46, p = 0.01)), transplantation seems to be worse (HR: 1.39, p = 0.05) but only in the (incorrect but commonly applied) model without time varying covariates. The long term (time depending) hazard ratio is shown to be virtually 1 and overall survival is virtually identical in both groups. Nonetheless no conclusion can be reached from a clinical point of view without assumptions which are by their very nature untestable unless all patients would be followed from diagnosis.

## Introduction

Myelodysplastic syndromes (MDS) constitute a heterogeneous group of clonal stem cell disorders characterized by hypercellular bone marrow, cytopenias and dysplastic cell features. Allogeneic stem cell transplantation from related or unrelated donors may cure patients with myelodysplastic syndromes [1–3]. However, due to the high treatment-related morbidity and mortality, this treatment approach is mainly performed in young patients with good performance status. Apart from the basic question whether a transplant should be performed at all, the optimal timing of transplantation is of great clinical interest but difficult to assess. Due to the lack of randomised trials, a retrospective comparison between transplanted patients and non-transplanted patients has to be performed. In order to avoid the inherent biases in non-randomized data as much as possible, investigators used a multi-state model. In this model, allogeneic stem cell transplantation maximized overall survival if stem cell transplantation is performed immediately after diagnosis for patients with IPSS intermediate 2 and high risk, while for patients with IPSS intermediate 1 and low risk delayed transplantation had maximal life expectancy [4].

Since MDS is a disease of elderly patients with a median age of more than 70 years at diagnosis, the majority of patients have been excluded from allogeneic stem cell transplantation option until recently. The reduction of therapy-related complications and the introduction of reduced-intensity conditioning regimens has increased the upper age limit and encouraging results of allogeneic stem cell transplantation in MDS-patients up to the age of 70 years have been reported [5–11]. However, whether elderly patients with MDS should undergo allogeneic stem cell transplantation is a matter of debate. We analysed, using a multi-state model with left and right truncation as well as time varying covariates, outcome of elderly advanced MDS patients (aged 55-69 years) with RAEB or RAEB-t who received either only best supportive care and were reported to the Düsseldorf registry (n = 137) or who received allogeneic stem cell transplantation and were reported to The European Group for Blood and Marrow Transplantation (EBMT) (n = 247).

### Design and Methods

This study aims at a comparison of two treatment modalities, one of which is transplantation, by analysing data from two separately obtained cohorts of patients. One cohort originates from a transplantation registry which only contains data from patients actually having received a transplant; the other cohort originates from a regional registry of patients not undergoing transplant. This study has a four-fold purpose [1]: to show that traditional statistical analysis approaches are inappropriate and biased [2]; to show that more advanced methods like multi-state models can aid in avoiding part of the bias inherent to an observational comparison [3]; to obtain an optimal estimate of the treatment effect from this data set to support the continuing clinical discussion on this topic; and [4] to show that even such an optimal estimate relies on implicit (clinical) assumptions that cannot be verified.

After having obtained optimal inference from this data set, some uncertainties still remain as to the amount of residual bias. This implies that differences between the two treatment modalities, in terms of overall survival from diagnosis, cannot be presented as sufficient evidence.

**Figure 1:**
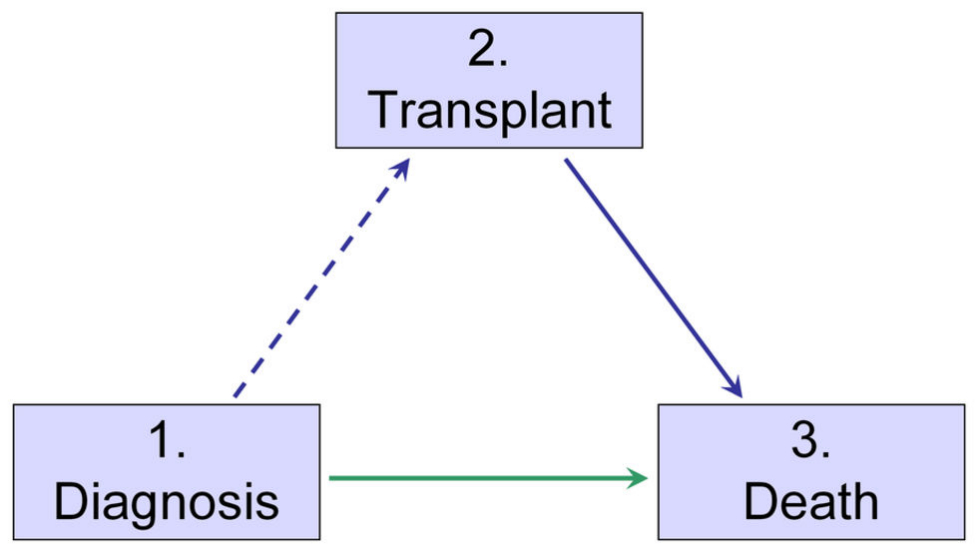
Multi-state model comparing transplant vs. non-transplant approach in elderly (55-69y) MDS patients.

The analysis of the actual data has been performed in a multi-state model. A multi-state model defines a number of states, based on clinically relevant stages in the disease/recovery process of the patient. Interest is in the rates of transitions between these states and how covariates influence these rates. For more details see 12. The multi-state model appropriate for our analysis is shown in Figure 1.

From the data the transition rates from states Diagnosis to Death can be estimated in the non-transplanted cohort with complete data from diagnosis onwards. The transition rate from Diagnosis to Transplant cannot be estimated directly since the transplant cohort does not contain patients who die while waiting for transplant (hence patients who would go into transition if they would have survived). The transition rate from Transplant to Death can be estimated by standard methods, provided left truncation is taken into account. The corresponding survival curve does estimate survival after transplant, but the curve is actually equivalent to a counterfactual survival curve in a cohort where each individual is immediately transplanted, a fact easily overlooked. Such a survival curve does not correspond to survival observed in any real-life cohort, where patients are not immediately transplanted and where as a result patients can also die before transplant.

If and only if one is willing to assume that at diagnosis patients with an intention-to-treat for transplant are on average identical to patients not elected to be transplanted, the death rate as a function of time elapsed since diagnosis can be estimated from the non-transplanted cohort. By applying this probability of dying before transplant to the transplanted cohort, one can reconstruct survival in the transplant cohort.

In the Appendix S1, by using simulation data, the naïve approach (i.e. direct comparison instead of multi state models) is shown to yield a severe bias in estimating survival from diagnosis. This affects both the comparison of standard Kaplan-Meier or Cox survival curves in the two cohorts, even while using left truncation to account for the unobserved period between diagnosis and transplant in the transplant cohort. The notion of left truncation is also referred to in the literature as “zero time shift bias” or “lead time bias” or “immortality time bias”.

More technical details on statistical and computational issues are also outlined in the Appendix S1. All computations related to the multi-state modelling approach were done using the mstate package [13] version 0.2.1 in R version 2.9.1.

### Data Sources

All outcome data were obtained from prospectively collected databases.

### Non-transplant cohort

Data from non-transplanted MDS patients with RAEB or RAEB-t were derived from the Düsseldorf registry. Only patients aged 55-69 years at diagnosis were selected. Patients who received transplantation were excluded. 137 patients were eligible for the analysis. Supportive care consisted of blood transfusions, growth factors, low dose cytosine arabinoside or in some cases intensive chemotherapy without transplantation. Diagnosis was RAEB in 100 and RAEB-t in 37 patients and the date of diagnosis was known in all cases (see table 1). The Düsseldorf registry is an MDS registry from a region in West-Germany which includes about 5 million inhabitants, data on morphology, treatment and death were reported on an annual basis.

**Table 1:** 

	**EBMT**				**Düsseldorf-Registry**	
	**(n = 247)**				**(n = 137)**	
	**at diagnosis**				**at diagnosis**	
**RAEB**	173 (70%)				100 (73%)	
**RAEB-t**	74 (30%)				37 (27%)	
	**at transplant**					
**RAEB**	135 (54%)					
**RAEB-t**	83 (34%)					
**transformed to sAML**	29 (12%)					
**Intensive chemotherapy before transplantation**						
**yes**	n=135						
**no**	n = 89					
**unknown**	n = 23					
**Status at transplantation**						
**CR**	n = 75					
**non CR**	n=149					
**missing**	n = 23					
**Patients sex**						
**male**	169 (68%)				83 (61%)	
**female**	78 (32%)				54 (39%)	
**Conditioning**						
**standard**	77 (31%)					
**reduced**	170 (69%)					
**Median age at diagnosis**	**58 years**				**62 years**	
**55-60**	167 (67%)				51 (37%)	
**60-69**	80 (33%)				86 (63%)	
**Cytogenetics known**	**n = 88**				**n = 79**	
**normal**	35 (40%)				40 (51%)	
**abnormal**	53 (60%)				39 (49%)	
**Percentiles of time from diagnosis to transplant in days**	1^st^	35	99^th^	10586		
	5^th^	73	95^th^	1395		
	10^th^	97	90^th^	1191		
	25^th^	141	75^th^	475		
	median	226				
**Time period diagnosis**	1971-2004				1998-2005	
**Time period transplant**	1998-2004 (50% in 2003 + 2004)					
**Donor**						
**HLA identical**	n = 176 (71%)					
**matched unrelated**	n = 71 (29%)					

### Transplant cohort

The transplanted patients were obtained from the EBMT database. 247 patients (male: n = 169, female: n = 78) were eligible for analysis. Disease status at diagnosis was RAEB (n = 173) and RAEB-t (n = 74). At time of transplantation diagnosis was RAEB (n = 135), RAEB-t (n = 83) and transformed secondary acute leukemia (n = 29). One- hundred- thirty five patients received induction chemotherapy and 75 achieved CR, while 1 achieved only PR and 20 relapsed or progressed after initial response and 37 patients were primary refractory and in 2 patients response status was not given, so overall at time of transplantation 75 patients were in CR, 149 in non-CR and in 23 patients information about pre-treatment were missing. All patients gave written informed consent for this study. The transplant cohort gave consent to The European Group for Blood and Marrow Transplantation (EBMT) for using their data for scientific purposes. As a Working Party of the EBMT (CLWP), we were allowed to use the data for this study. The non-transplant cohort gave consent to Ulrich Germing (coauthor) for using their data for scientific purposes. All data were analyzed anonymously. For details see table 1.

## Results

### Basic description

The median follow-up among survivors in the non-transplant cohort was 11 months with a maximum follow-up of 95 months. Median follow-up among survivors of the transplant cohort measured from diagnosis was 56 months (with a maximum of 132 months), while measured from transplant it was 42 months (with a maximum of 109 months). Median time between date of diagnosis and date of transplant observed in the transplant cohort was 7 months (survivors and deaths together). The cumulative incidence of non-relapse mortality of the transplant cohort at 1 year and 3 years post-transplant was 15% and 31%, respectively. The cumulative incidence of relapse was 14% and 29% at the same time points. The 5 year estimated overall survival from diagnosis was 23% (95% CI: 12-34) for the non-transplant cohort; 5 year estimated overall survival from transplant was 31% (95% CI 25-38) for the transplant cohort.

The interested reader is referred to the Appendix S1 where we show that a number of naïve (and commonly applied) methods will estimate the wrong parameters and would possibly yield a (highly) biased interpretation of the data and that the multi-state approach allows in principle for a correct interpretation.

From the Appendix S1 we may infer that a multi-state approach will yield an unbiased estimate of the treatment effect (i.e. transplant versus non-transplant) if the death rate in both populations is the same before the transplant (as assumed by the so-called Markov model).

In our study, we can only assume that the rates are the same. This in turn is only true if indeed the decision to go for transplant is not correlated with any patient characteristic which itself is correlated with the probability of mortality. In a randomized study this would be guaranteed by the randomization procedure; in our study it can only be approximated by correction for status/stage related factors at diagnosis, age of the patient etc. and knowledge about the reasons for (not) attempting a transplant as a treatment modality.

### Comparison of treatment modalities

**Figure 2:**
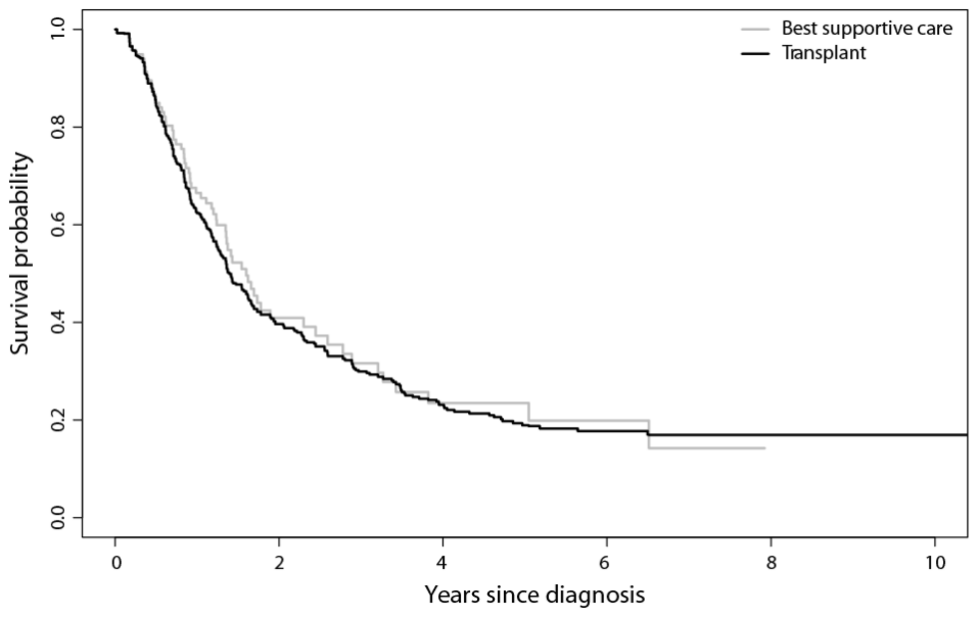
Estimated Kaplan-Meier survival curves for transplant and non-transplant cohorts, derived from a multi-state model.

Applying the multi-state method, the overall (average) hazard ratio of the transplant group versus best supportive care, assuming proportional hazards, is estimated as 1.29 (95% CI: 0.95-1.76; p = 0.11). After adjustment for year of diagnosis as a continuous variable, the hazard ratio is the same 1.28 (95% CI: 0.94-1.74; p = 0.12). There was no evidence that the effect of year of diagnosis differed between Düsseldorf registry and EBMT database (p = 0.79) The median overall survival from diagnosis for the non-transplant and the transplant cohort was virtually identical: 19 months, (see Figure 2).

From Figure 2 we may infer that both populations, given the (unknown) selection mechanisms underlying the selection of patients for (non-)transplant, both groups have the same survival. Note that the usual steep descend of the curve among transplanted patient directly after transplant due to TRM is entirely invisible since the curves start at diagnosis and all transplanted patients start their period where they are at risk for TRM, at a different point in time (spreading this usually steep descent over a long period “after diagnosis” compared to a relatively short period “after transplant”) which makes this pattern almost invisible.

In a multivariate analysis and assuming proportional hazards (which turns out to be an incorrect assumption as discussed below), adjusting for age at diagnosis, sub-diagnosis (RAEB vs. RAEB-t), cytogenetics (abnormal vs. normal), the hazard ratio for transplantation was HR: 1.37 (95% CI: 1.00-1.89; p = 0.05); for age > 60: HR 1.43 (95% CI: 1.07-1.91; p = 0.02), for RAEB-t: HR 1.03 (95% CI: 0.78-1.37; p = 0.84), and for abnormal cytogenetics: HR 1.35 (95% CI: 1.00-1.81; p = 0.05)

**Figure 3:**
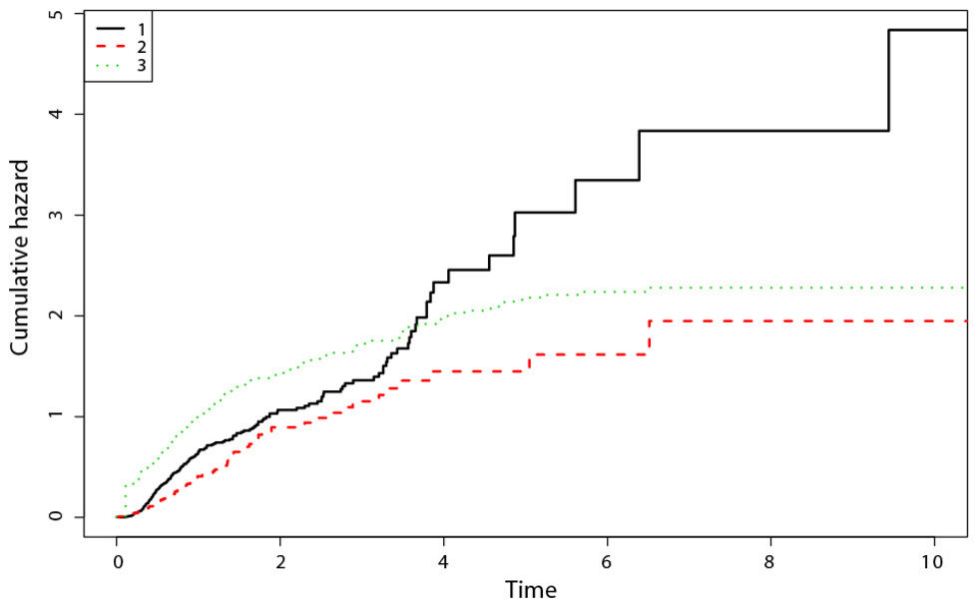
Estimated cumulative transition rates derived from a multi-state model (1: Diagnosis-Transplant; 2: Diagnosis-Death if not transplanted; 3: Transplant-Death); time in years.

Corresponding to this multivariate analysis belongs a graph estimating the transition rates to death in both groups as well as the transition rate from diagnosis to transplant (see Figure 3).

Horizontally the time axis measures time in years. The vertical axis depicts the accumulated average rate (up to a certain point in time) at which patients’ transit from one state to the other. The absolute height of the curve is of no importance for our discussion. It is the shape that counts for the clinical interpretation:

The highest curve (1) depicts the cumulative transplantation rate (estimated under right truncation (using the method proposed by[14]) since diagnosis as a function of the number of years elapsed since diagnosis. The curve is approximately a straight line indicating that transition to transplant is almost uniformly happening over time.

The lowest curve (2) is the mortality hazard in the non-transplant group, measured in years since diagnosis and (3) the mortality rate in the transplanted group measured in years since transplant. Both curves flatten towards the end of the horizontal axis, indicating a tendency of the underlying survival curve to become “horizontal”. Clearly the death rate in the transplanted group is much higher very shortly after diagnosis, but one should bear in mind that TRM is an inherent phenomenon among transplanted patients shortly after transplant and those patients transplanted shortly after diagnosis therefore exhibit mortality also shortly after diagnosis as a result of the procedure and not necessarily as a result of the short interval between diagnosis and transplant as a clinical underlying risk factor. This phenomenon leads to the overall (and misleading) estimate of a detrimental effect of transplantation as an average hazard ratio (1.29 univariately or 1.37 multivariately) when using a simple but inappropriate Cox model.

However, because of Treatment Related Mortality and the broad range of periods between diagnosis and transplant, the hazard ratio is not constant at all but varies significantly over time.

In other words: while the notion of transplant related mortality measured since “transplant” occurs in a fairly narrow time range, the same phenomenon becomes more dispersed over time when calculated from diagnosis and thus could easily be misinterpreted as a continuing excess risk among transplanted patients when looking at survival curves starting at diagnosis.

The following picture shows an estimate of this hazard ratio (on a log scale so the vertical zero corresponds to a hazard ratio of 1). The horizontal axis measures time in years since diagnosis (on a non-linear scale).

**Figure 4:**
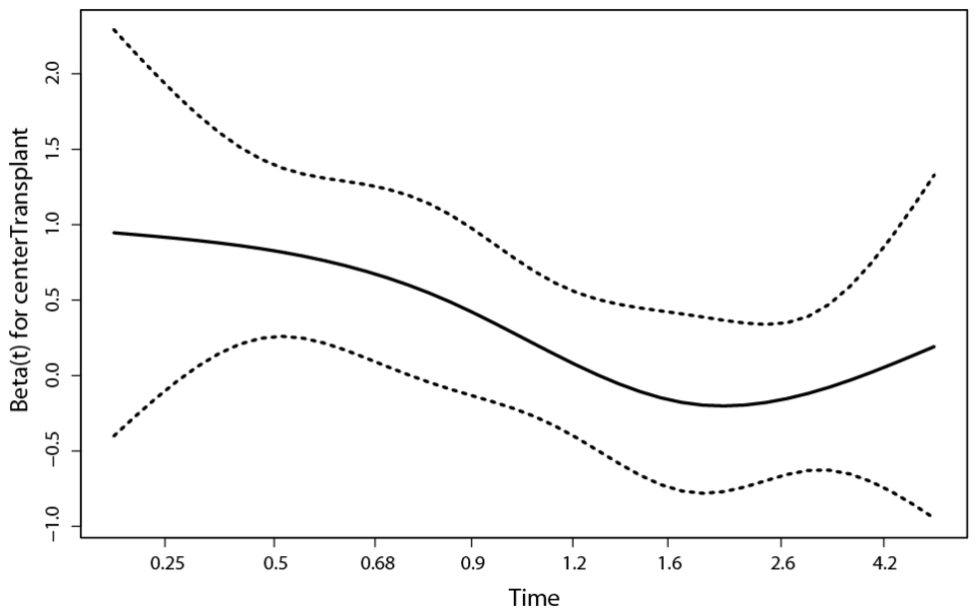
Estimated log(hazard ratio) comparing the transplant to the non-transplant cohort in a multi-state model using a time-varying factor for the treatment effect, together with a 95% confidence interval; the time is in years.

Here we see from Figure 4 that the entire detrimental effect of transplant when estimated as one average over the entire follow up time, is generated in the first year after diagnosis while thereafter the Hazard Ratio is 1 (log(HR)=0). The null hypothesis that the hazard ratio is constant over time is rejected (p= 0.028; for the test description see 15).

Further detailed analysis leads to an estimated HR of 5.8 during the first 3 months after diagnosis and a HR of almost exactly 1 after a year.

In our population with age above 55, the interval between diagnosis and transplant turns out not to be a risk factor at all (after transplant). Although in the entire transplanted population in the EBMT registry, the interval is indeed an adverse risk factor, this well-known phenomenon turns out to hold only for the younger patients: a strong and significant interaction between age at transplant and interval between diagnosis and transplant is indeed present in The EBMT Registry (data not shown) and actually totally removes the supposed effect of the interval in older patients.

## Discussion

In the current study we attempted to answer the clinically relevant question whether elderly patients (55-69 years) with advanced MDS (RAEB and RAEB-t) may benefit from allogeneic stem cell transplantation in comparison to a non-transplant approach. We therefore collected data from RAEB and RAEB-t patients who were registered either in a non-transplant registry (Düsseldorf-Registry) or in a transplant registry (EBMT-registry). Despite including several statistical methods such as multi-state models, left and right truncation, not all potential biases can be excluded and the analysis is still based on (untestable or even likely to be untrue) assumptions.

First, transplant patients are registered only at time of transplantation, which means death before transplantation is not possible; therefore the death rate before transplantation has to be estimated from the non-transplanted patients in the Düsseldorf registry and both the time from diagnosis to transplant and from transplantation to death have to be estimated taking into account right and left truncation respectively.

Secondly, an untestable (and unlikely to be true) assumption is that the transplant and non-transplant group are comparable with respect to risk factors at diagnosis. Since IPSS scoring was not possible for both groups due to the lack of cytogenetic data, we included only patients with advanced RAEB and RAEB-t MDS to create comparable groups. However, this does not exclude unmeasured selection bias for the transplant cohort, since from diagnosis to transplant (median 7.4 months) 16% progressed to secondary acute leukemia or RAEB-t.

An approach similar to ours using a Markov model with piecewise constant transition rates has been used by the CIBMTR comparing (mainly younger) patients with MDS who received a HLA identical sibling transplantation after standard myeloablative conditioning with a cohort of patients without transplant (4). They found for low and intermediate risk patients a delayed transplantation is advantageous, while for patients with intermediate 2 and high risk according IPSS immediate stem cell transplantation is associated with a maximal life expectancy.

Obviously optimal timing of transplant depends on the differences in death rates between the non-transplant and the transplant cohort (the transplant effect) over time. In our data we found a transplant effect that on average was in favor of the non-transplant cohort over the whole follow-up period. However, this average is not only an average over follow-up time but also an average over all possible intervals between diagnosis and transplant in our data; moreover the effect is a reflection of current transplant practice correlated with risk factors observed at diagnosis but lacking among those dying before transplant.

This all implies that the outcome of a scenario analysis as was performed in Cutler et al. [4] evaluating the effect of specific assumptions on the timing of the transplant procedure after diagnosis can only be done by modelling the effect of the interval both before and after the transplant as well as looking at possibly non-constant hazard ratio of transplant with respect to death. If the interval-effect (between diagnosis and transplant) would in reality be non-existent for survival after transplant, the result would be rather trivial: since in these models the death rate after diagnosis but before transplant is estimated from the non-transplanted cohort, evidently both cohorts should have more similar outcome the longer this waiting time is assumed to be, forcing the hazard ratio towards 1. A short waiting time applies the death rate after transplant very quickly after diagnosis; a long waiting time assumes a death pattern equal to nontransplanted patients for an extended period of time since diagnosis.

One should note that “early after diagnosis” is identical to “early after transplant” for those patients transplanted shortly after diagnosis. Transplanting patients shortly after diagnosis will inevitably “generate” mortality, not because an early transplant is necessarily bad (it might even reduce mortality) as such but simply because mortality will be associated with the transplant procedure anyhow and the earlier transplant is scheduled, the earlier mortality occurs when measured “since diagnosis”.

Hence in our opinion still no conclusion about the causal effect of the interval between diagnosis and transplant can be drawn from observational data, no matter how sophisticated these models are, if sufficient covariates at diagnosis for patients dying between diagnosis and transplant are lacking. Obviously the group of transplant patients contains patients with different levels of risk, according to their CR-status but also according to the administration of intensive chemotherapy or their resistance against chemo. We stress the fact that there is not sufficient information in the data to rule out an appreciable selection bias which could invalidate the crucial assumption of equal mortality risk in both groups.

More generally speaking we have serious doubts whether such conclusions can be drawn from such observational data at all. There seems to be an unavoidable bias in data which lack information at diagnosis as well as information on death rates in the pre-transplant period, caused also by the correlation between the phenomenon of unavoidable TRM and the fact that “early after transplant” is the same as “early after diagnosis” for patients transplanted quickly after diagnosis. Disentangling this correlation requires data which are not present. We therefore express the opinion that this crucial assumption of equal death rates plays an equally essential part in the Cutler paper without being made entirely explicit ( [4], page 548). Differences in approach between the Cutler paper and ours are in essence small: we use non-parametric or semi-parametric models, Cutler uses parametric ones; we emphasize that you need to know the death pattern while waiting for an intended transplant; we emphasize that unverifiable assumptions are needed also in the Cutler approach. We can see no reason why the approach in the Cutler paper, just like in our approach, could lead to conclusions about the effect of the interval between diagnosis and transplant without the aforementioned basic, unverifiable assumptions. This is not a matter of being right or wrong for any of these approaches: they just rely on crucial *assumptions*.

Moreover, even with an optimal choice of statistical methodology, no valid analysis can be done without the availability of a comprehensive dataset including all variables with solid prognostic significance (such as IPSS-R).

In our analysis, an early disadvantage for stem cell transplantation was probably by the non-relapse mortality of the transplant cohort which exceeded 30% at 3 years. The non-relapse mortality has been reduced in the more recent years by reducing risk of severe GvHD and lowering the therapy-related toxicity of the conditioning regimen [5,6,8-11]. Furthermore, if no HLA-identical sibling is available a careful donor selection by high resolution HLA-matching lowers the risk of non-relapse mortality [16]. More recently hypomethylating agents in MDS patients have been shown to prolong transformation to AML and survival in comparison to “best supportive care” and have been approved for treatment of high risk MDS [17]. In our non-transplant cohort none of the patients received hypomethylating agents. A prospective trial comparing hypomethylating agents with a reduced-intensity transplant approach according to donor availability is needed and has been recently started as EBMT labelled trial in The German MDS Study Group (NCT 01404741).

Finally, we would like to put forward our conclusion with respect to the clinical target we tried to achieve, based on the arguments put forward in this paper:

In order to obtain a reliable and useful answer to the effect of the transplant, including the timing relative to the time of diagnosis, a randomized clinical trial is absolutely necessary. Such a trial should have both a randomization structure that allows for a useful clinical interpretation as to the decision and timing of an “intention to go for transplant” and should incorporate both patient and clinic based parameters. Especially the timing of transplant should be captured in the design of the trial, for example by stratification or by multiple randomizations in time. Unlike many other situations in which observational data, if carefully modelled, could reach evidence levels almost comparable to clinical trials, this is clearly not easy in the framework of transplantation, unless of course a register of patients from diagnosis onwards would be available with a sufficiently rich information infrastructure to capture all covariates correlated with the decision to go for transplant. The latter is clearly a Utopia.

In conclusion we will have to use clinical trials to define and assert the position and timing of the transplant procedure among other treatment options since even an assertion that allogeneic SCT is at least equivalent to the best alternative therapy in a specific subgroup of patients cannot be substantiated, not even by the best statistical methods available today.

## Supporting Information

Appendix S1 **Using simulated survival data to show inappropriateness of standard methods when comparing transplanted and non-transplanted patients.**
(DOCX)

Appendix S2 **Participating EBMT Centers.**
(DOCX)

Figure S1 (a) SIMULATED DATA: Survival curve assuming constant death rate before transplantation (see text). b: SIMULATED DATA: Survival curve of transplant vs. best support care (naïve analysis) (see text). c: SIMULATED DATA: Survival curves taking left truncation into account (dark solid: left truncation; dark dashed: complete cohort). d: SIMULATED DATA: Survival curves after multi-state modelling, perfectly recapturing the structure of the simulated data (dark solid: left truncation; dark dashed: complete cohort).
(TIF)
