# Supplementary material for: Comparison of Allogeneic Stem Cell Transplantation and Non-Transplant Approaches in Elderly Patients with Advanced Myelodysplastic Syndrome: Optimal Statistical Approaches and a Critical Appraisal of Clinical Results Using Non-Randomized Data
Source: PLoS One. 2013 Oct 7;8(10):e74368. doi: 10.1371/journal.pone.0074368 (PMC3792099; doi:10.1371/journal.pone.0074368)
Supplement: Appendix S1 — Using simulated survival data to show inappropriateness of standard methods when comparing transplanted and non-transplanted patients. (DOCX) [file pone.0074368.s001.docx]

**Appendix S1: Using simulated survival data to show inappropriateness of standard methods when comparing transplanted and non-transplanted patients**

**Biased results from a naïve approach and justification of multi-state modelling through *simulation* of data**

Direct comparison of two cohorts, one transplanted and one not-transplanted, is methodologically wrong. Patients in any transplanted cohort must be alive after diagnosis in order to be transplanted. Hence all patients who were scheduled for transplant and died between diagnosis and the intended transplant date are not available and therefore any Transplant Registry is not representative of an “intent-to-treat” population of patients scheduled for transplant, while the non-transplanted cohort is indeed followed from diagnosis onwards. Statistically this phenomenon is called left-truncation. Therefore the survival curve from Transplant to Death is well-defined but what needs to be estimated is the survival curve from Diagnosis to Death.

We simulated data with the following properties (just as an example, not attempting to mimick the actual disease and treatment progression in our actual data!): random allocation to the two treatment modalities (transplant or no-transplant); two groups of 500 patients each; a (constant) death rate of 0.3 per year after diagnosis but before transplant (if transplanted); a constant death rate of 0.3*HR after transplant (for simplicity’s sake HR= hazard ratio)); a transplantation rate of 0.4 per year after diagnosis; finally a constant independent censoring rate of 0.25 per year. In order to illustrate the issues a rather extreme hazard ratio of 0.4 was chosen.

First simulation analysis: complete data on all patients (which is not available in our actual study data since we do not observe patients who die before transplant while being allocated to that subgroup). The survival curve of the transplant cohort will serve as benchmark for future analyses. The difference in survival in favour of transplant is clear. See figure S1a.

Note that the difference between the survival curves is not as large as the hazard ratio of 0.4 between death rates after and before transplant would suggest. The reason is that in the complete transplant intention-to-treat cohort a substantial proportion of patients also die before transplant (at the pre-transplant death rate).

Second simulation analysis: naïve analysis of observed data (the dead patients in the intent-to-transplant cohort are removed just as they would not be present in any Registry collecting data on actually transplanted patients): See figure S1b.

Clearly the transplanted patients appear to have better outcome just by removal of the unobserved dead patients: survival for the transplanted patients and hence the transplant effect is severely overestimated.

Third simulation analysis: taking into account left-truncation.

In this analysis one takes into account that transplant patients were not observed to be at risk for death until transplant. The corresponding survival curve of the transplant group is a consistent estimate of a counterfactual (imaginary) survival curve where each patient in the transplant cohort is transplanted immediately after diagnosis. It does not correspond to survival in a cohort where patients are not immediately transplanted and hence may also die before transplant. Figure 5c shows the survival estimate based on left truncation (dark solid line) and the target survival estimate based on the complete cohort from figure S1a (dark dashed line).

The solid survival curves compare survival before and after transplant; the transplant survival curve does not correspond to what one would observe in a transplant cohort followed from diagnosis since it inherently assumes that no selection takes place between diagnosis and transplant.

The survival curve that we would like to estimate for the transplant group is the survival in a cohort of patients, followed from diagnosis, who are not transplanted immediately, and who may also die before they could be transplanted. This “truth” can only really be evaluated in a randomized clinical trial; with only data from a transplant registry at our disposal the best we can do is to approximate it by modelling, based on assumptions about the underlying death pattern in the transplanted group. The real pre-transplant death rate in the transplant cohort cannot be estimated from the (unobserved!) data.

Finally, a calculation (see [1], a tutorial by Putter 2007) combining all three transition intensities in the multi-state model (see also figure 1 in the main part of this paper), using as underlying death rate among transplanted patients before their transplant that from the subgroup of non-transplanted patients, will recapture the underlying survival curves: See figure 1d-S. This calculation uses the Markov assumption. The transition rate from diagnosis to transplant was estimated taking right truncation into account using the methods of Gill and Keiding [2].

The above model shows that the multi-state approach will be unbiased IF the death rate in both populations is the same before the transplant. For the purpose of our example, this is assured by our simulation of the data.

However in our “real life data” study, we can only *assume* that the rates are the same. This in turn is only true if indeed the decision to go for transplant is not correlated with any patient characteristic which itself is correlated with the probability of mortality! In a randomized study this would be guaranteed by the randomization procedure; in our study it can only be approximated by correction for status/stage related factors at diagnosis, age of the patient etc and knowledge about the reasons for (not) attempting a transplant as treatment modality.

Therefore it is crucial to ascertain the comparability of the two cohorts at baseline, i.e. at diagnosis. Without that comparability also the multi-state approach would be biased.

This ascertainment will be addressed after presentation of the final results of our analysis of the true data.

In order to avoid a misunderstanding in the interpretation of these curves, we would like to stress that all curves originate at diagnosis and the notion of “transplant related mortality” which forces survival curves to steeply drop shortly after time zero (=diagnosis) when relatively few patients start being at risk (since diagnosis) for “death after transplant”, is a phenomenon we did not incorporate in our simulated data but which could be present in real study data leading to wide confidence intervals at the start.

**References**

1 Putter H, Fiocco M, Geskus RB. (2007) Tutorial in biostatistics: Competing risks and multi-state models. Stat Med 26: 2389-2430.

2 Gill RD, Keiding N. (1990) Random truncation models and Markov processes. Ann Statist 18: 582-602.
