# Supplementary material for: Comparison of Allogeneic Stem Cell Transplantation and Non-Transplant Approaches in Elderly Patients with Advanced Myelodysplastic Syndrome: Optimal Statistical Approaches and a Critical Appraisal of Clinical Results Using Non-Randomized Data
Source: PLoS One. 2013 Oct 7;8(10):e74368. doi: 10.1371/journal.pone.0074368 (PMC3792099; doi:10.1371/journal.pone.0074368)
Supplement: Appendix S2 — Participating EBMT Centers. (DOCX) [file pone.0074368.s002.docx]

**Appendix S2**

Participating EBMT Centers:

| control group from Germany, via Düsseldorf | 137 |
| --- | --- |
| D.Niederwieser, University of Leipzig, Leipzig, Germany [389] | 14 |
| J. Finke, University Hospital, Freiburg, Germany [810] | 11 |
| G.Mufti, GKT school of Medicine London, United Kingdom [763] | 10 |
| M. Bornhauser, Universitätsklinikum Dresden, Dresden, Germany [808] | 8 |
| L.Bergmann, Universität Ulm, Ulm, Germany [204] | 7 |
| M.Falda, Azienda Ospedaliera S. Giovanni, Torino, Italy [231] | 7 |
| A.Zander, University Hospital Eppendorf , Hamburg, Germany [614] | 7 |
| J.Sierra, Hospital Santa Creu I Sant Pau, Barcelona, Spain [260] | 6 |
| N. Milpied, Hopital Haut-Leveque, Pessac, France [267] | 6 |
| A.Gratwohl, Kantonsspital, Basel, Switzerland [202] | 5 |
| J.Cornelissen, D.den Hoed Cancer Centre/AZR, Rotterdam, The Netherlands [246] | 5 |
| D.Beelen, University Hospital, Essen, Germany [259] | 5 |
| J.P.Jouet, Hopital Claude Huriez, Lille, France [277] | 5 |
| T.Ruutu, Helsinki University Central Hospital, Helsinki, Finland [515] | 5 |
| N.Russell, Nottingham City Hospital, Nottingham, UK [717] | 5 |
| M.Boogaerts, University Hospital of Leuven, Leuven, Belgium [209] | 4 |
| A.Bacigalupo, Ospedale San Martino, Genova, Italy [217] | 4 |
| R.Haas, Heinrich Heine Universität, Düsseldorf ,Germany [390] | 4 |
| D.Selleslag, A.Z. Sint-Jan, Brugge, Belgium [506] | 4 |
| H.Schouten, University Hospital Maastricht, Maastricht, The Netherlands [565] | 4 |
| Y. Beguin, University of Liege, Liege, Belgium [726] | 4 |
| D.Caballero, Hospital Clinico, Salamanca, Spain [727] | 4 |
| E.Gluckman, Hopital St. Louis, Paris, France [207] | 3 |
| D.Blaise, Institut Paoli Calmettes, Marseille, France [230] | 3 |
| T. de Witte, Univ.Med.Cent.St. Radboud, Nijmegen, The Netherlands [237] | 3 |
| A.Torres Gomez, Cordoba Hospital – Reina Sofia, Córdoba, Spain [238] | 3 |
| V.Leblond, Pitie-Salpetriere, Paris, France [262] | 3 |
| G.Gastl, University Hospital Innsbruck, Innsbruck, Austria [271] | 3 |
| A.Fauser, Klinik für K.M.T. & Hämato-Onkologie Idar-Oberstein, Germany [592] | 3 |
| J.M.Ribera Santasusana, Hosp.Univ. Germans Trias I Pujol, Barcelona, Spain [613] | 3 |
| A.Vitek, Inst. of Hematology and Blood Transf., Prague, Czech Republic [656] | 3 |
| M.Sanz, Hospital Universitario La Fe,Valencia, Spain [663] | 3 |
| P.Ljungman, Huddinge University Hospital, Huddinge, Sweden [212] | 2 |
| S.Mackinnon, Royal Free Hospital and School of Med., London, UK [216] | 2 |
| E. Petersen, University Medical Centre Utrecht, Utrecht, The Netherlands [239] | 2 |
| P. Di Bartolomeo, Ospedale Civile, Pescara, Italy [248] | 2 |
| T.Littlewood, The Oxford Radcliffe Hospital, Oxford, UK [255] | 2 |
| S.McCann, St. James Hospital Trinity College, Dublin, Ireland [257] | 2 |
| W.Siegert, Charite-Virchow Klinikum d.Humboldt-Univ., Berlin, Germany [293] | 2 |
| P.Zachée, AZ Stuivenberg, Antwerp, Belgium, [339] | 2 |
| N. Gratecos, Hôpital de l'Archet I, Nice, France [523] | 2 |
| G. Doelken, Ernst-Moritz-Arndt Univ. Greifswald, Germany [530] | 2 |
| F.Narni, Univ. Modena, Policlinico, Modena, Italy [543] | 2 |
| R.Marcus, Addenbrookes Hospital, Cambridge, United Kingdom [566] | 2 |
| G.Ossenkoppele, Free University Hosp. A’dam, Amsterdam, The Netherlands [588] | 2 |
| JH.Bourhis, Institut Gustave Roussy, Villejuif, France [666] | 2 |
| L.Jebavy, Charles Univ. Hosp. Hradec Králové, Czech Republic [729] | 2 |
| G.Juliusson, University Hospital Linköping, Linköping, Sweden [740] | 2 |
| A.Nagler, Tel-Hashomer, Tel Aviv, Israel [754] | 2 |
| R. Willemze, Leiden University Hospital, Leiden, The Netherlands [203] | 1 |
| C.Heilmann, Rigshospitalet, Copenhagen, Denmark [206] | 1 |
| D.Bron, Institut Jules Bordet, Brussels, Belgium [215] | 1 |
| J.Ledermann, University College London Hospital, London , United Kingdom [224] | 1 |
| A. Ferrant, Cliniques Universitaires St. Luc, Brussels, Belgium [234] | 1 |
| L.Brinch, Rikshospitalet, The National Hospital, Oslo, Norway [235] | 1 |
| J.Fernandez-Ranada, Hospital de la Princesa, Madrid, Spain [236] | 1 |
| C.Cordonnier, Hopital Henri Mondor, Creteil, France [252] | 1 |
| M. Mohty, Hotel Dieu, Nantes, France [253] | 1 |
| M.Gramatzki, Univ. Hosp. Schleswig-Holstein, Kiel, Germany [256] | 1 |
| S.Slavin, Hadassah University Hospital, Jeruzalem, Israel [258] | 1 |
| F.Guilhot, Hopital La Miletrie, Poitiers, France [264] | 1 |
| G.Lambertenghi Deliliers, Ospedale Maggiore di Milano, Milano, Italy [265] | 1 |
| E.Kansu, Hacettepe University, Ankara, Turkey [292} | 1 |
| E.Morra, Ospedale di Niguarda Ca’Granda, Milano, Italy, [294] | 1 |
| P. Coser, Hospital San Maurizio, Bolzano, Italy [299] | 1 |
| A.Bosi, Ospedale di Careggi, Firenze, Italy [304] | 1 |
| W.Linkesch, Karl Franzens University Graz, Graz, Austria [308] | 1 |
| F. Lauria, Policlinico Le Scotte, Siena, Italy [321] | 1 |
| D.Culligan, Grampian University Hospitals Trust, Aberdeen, Scotland, UK [344] | 1 |
| S.Robinson, Bristol Oncology Centre, Bristol, United Kingdom [386] | 1 |
| J.Ortega, Hospital M. Infantil Vall d’Hebron, Barcelona, Spain [527] | 1 |
| E.Pogliani, Ospedale San Gerardo, Monza, Italy [544] | 1 |
| A. Fassas, G.P. Gen. Hosp. of Thessaloniki, Exokhi, Greece [561] | 1 |
| W. Knauf, Klin. Benjamin Franklin, FU Berlin, Berlin, Germany [590] | 1 |
| J.Vorlicek, University Hospital Brno, Brno, Czech Republic [597] | 1 |
| A. Bosi, Ospedale di Careggi, Firenze, Italy [304] | 1 |
| N. Harhalakis, Evangelismos Hospital, Athens, Greece [622] | 1 |
| F.Benedetti, University of Verona, Verona, Italy [623] | 1 |
| H.Wandt, Klinikum Nürnberg, Nürnberg, Germany [625] | 1 |
| D.Carrera Fernandez, Hospital Covadonga, Oviedo, Spain [642] | 1 |
| L.Gugliotta, Arcispedale S.Maria Nuova, Reggio Emilia, Italy [660] | 1 |
| E.le Gall, CHRU de Rennes-Hôpital Sud Clinique Med. Infantile, Rennes, France [661] | 1 |
| M. Boccadoro, Ematologia Universitaria, Torino, Italy [696] | 1 |
| R.Hermann, Royal Prth Hospital, Perth Western Australia, Australia [710] | 1 |
| V.Koza, Charles University Hospital, Pilsen, Czech Republic [718] | 1 |
| J.Hansz, K. Marcinkowski University of Medical Science, Poznan, Poland [730] | 1 |
| F. Hernandez, Hosp. Univ. La paz, Madrid, Spain [734 | 1 |
| S.Amadori, Univ.Tor Vergata, St.Eugenio Hospital, Rome, Italy [756] | 1 |
| A.Newland, St.Batholomew’s and the Royal London Hospital, London, UK [768] | 1 |
| A. Angelucci, Osp. 'A. Businco', Cagliari, Italy [791] | 1 |
| N.Patton, Canterbury Health Laboratory, Christchurch, New Zealand [798] | 1 |
| C.Bordignon, Isitituto Scientifico H.S.Raffaele, Milano, Italy [813] | 1 |
| G.Mariani, Univ.di Palermo, Palermo, Italy [814] | 1 |
| J.L.Diez-Martin, Hospital G.U. Gregorio Maranon, Madrid, Spain [819] | 1 |
| M.Hamon, Plymouth Hospitals NHS Trust/Derriford Hospital, Plymouth, UK [823] | 1 |
| A. Levis, H SS. Antonio e Biagio, Alessandria, Italy [825] | 1 |
| H.Tilly, Centre Henri Becquerel, Rouen, France [941] | 1 |
