# Supplementary figures and images for: Comparison of Allogeneic Stem Cell Transplantation and Non-Transplant Approaches in Elderly Patients with Advanced Myelodysplastic Syndrome: Optimal Statistical Approaches and a Critical Appraisal of Clinical Results Using Non-Randomized Data
Source: PLoS One. 2013 Oct 7;8(10):e74368. doi: 10.1371/journal.pone.0074368 (PMC3792099; doi:10.1371/journal.pone.0074368)

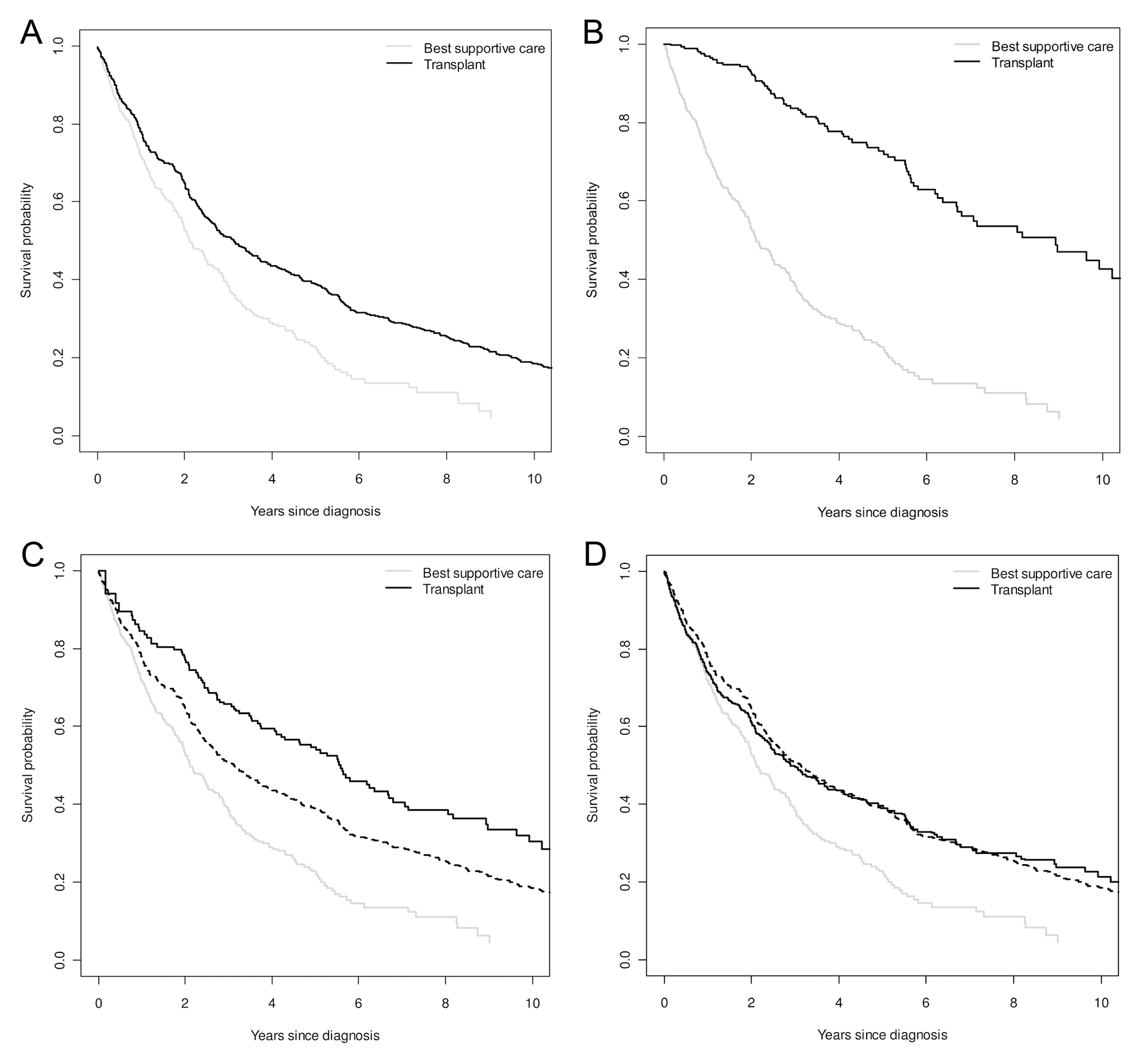

Supplement: Figure S1 — (a) SIMULATED DATA: Survival curve assuming constant death rate before transplantation (see text). b: SIMULATED DATA: Survival curve of transplant vs. best support care (naïve analysis) (see text). c: SIMULATED DATA: Survival curves taking left truncation into account (dark solid: left truncation; dark dashed: complete cohort). d: SIMULATED DATA: Survival curves after multi-state modelling, perfectly recapturing the structure of the simulated data (dark solid: left truncation; dark dashed: complete cohort). (TIF) [file pone.0074368.s003.tif]
